# Emerging competencies for logistics professionals in the digital era: A literature review

**DOI:** 10.3389/fpsyg.2022.965748

**Published:** 2022-10-25

**Authors:** Le Yi Koh, Kum Fai Yuen

**Affiliations:** School of Civil and Environmental Engineering, Nanyang Technological University, Singapore, Singapore

**Keywords:** digitalization, Industry 4.0, COVID-19, emerging competencies, logistics professionals

## Abstract

The speed of technology integration among businesses has accelerated during the COVID-19 pandemic due to the work-from-home arrangements and safe distancing regulations, prompting businesses to automate operations and digitalize work environments. These impacts have disrupted work environments and operational processes, and a fresh set of competencies is required to stay competent in this new normal. Consequently, there is a need to develop a state-of-the-art competency framework for logistics professionals during these trying times. This study has adopted the Preferred Reporting Items for Systematic Reviews and Meta-Analyses to review, identify, and update the emerging competencies required by logistics professionals. The relevant academic documents were narrowed down to 81 and were used to identify the emerging competencies relevant to Industry 4.0 and COVID-19. The competencies were subsequently categorized into four key domains i.e., business, logistics, digital, and personal competencies, with a total of 17 sub-domains. This state-of-the-art framework contributes to academic research by updating the existing competency frameworks. Future research can also build upon this holistic list of emerging competencies by utilizing it to reduce the competency gaps faced by those who are less technology savvy i.e., older logistics professionals. Additionally, future research can correlate the competency framework to organizational learning theories to improve the overall performance of logistics companies.

## Introduction

Logistics facilitates efficient and effective product flows from the point of origin to the point of consumption, which in turn fulfills customer demands and supports supply chains (Lummus et al., 2001). In 2011, Industry 4.0 began gaining traction and kickstarted the digital economy (Schmidtke et al., 2018). For the logistics sector, Industry 4.0 and its information and communication technologies (ICT) digitalized freight transportation, paving the way for real-time tracking of cargoes, predictive analytics, and automated document flows (Korepin et al., 2020).

Fast forward to the present day, the COVID-19 pandemic has spurred governments worldwide to implement lockdown measures to curb the virus. Although logistics companies were deemed as essential and permitted to continue operations, there were safe-distancing regulations and quotas allowing only a certain number of professionals in workplaces (Edwards, 2020). This incentivized logistics companies to invest in autonomous delivery robots, automated warehouses, and autonomous mining technologies (Nobre, 2020). With COVID-19 accelerating digitalization (Chen et al., 2020), traditional logistics activities such as logistics communication, material handling, and order processing are digitalizing as well. Consequently, the required competencies e.g., skills, knowledge, and capabilities are evolving and must be identified for logistics professionals to continue contributing to their work environments.

Despite the importance of logistics professionals to global supply chains in the digital era, the most recent studies on Scopus focused on management issues such as green logistics, reverse logistics, service quality, and Industry 4.0 implications, rather than the competencies of logistics professionals. Even among the studies that addressed competencies, most highlighted the role of Institutes of Higher Learning in developing digital literacy but did not identify the specific digital competencies required by aspiring logistics professionals (Cherniavskyi et al., 2019; Scherbakov and Silkina, 2019; Korepin et al., 2020). Thus, this study examined existing competency frameworks to better identify the relevant competencies for logistics professionals in the digital era. Cantoni and Bisogni (2019) utilized the European Logistics Association Qualification Framework to examine the competency of logistics professionals, but these standards are not sufficient as they do not cover upcoming issues like resilience, sustainability, and digitalization. In another study, Heaslip et al. (2019) utilized the humanitarian logistics competency framework to identify 29 competency categories for humanitarian logisticians, but only two categories were related to ICT as the rest consisted of technical and management-related domains. Another frequently discussed framework was the business, logistics, management (BLM) framework developed by Poist (1984) for entry-level and senior-level logistic professionals. Over the years, it has been subjected to extensions to better reflect the competitive environment. For example, Sangka (2017) extended the BLM framework with the ICT domain to reflect the technological advancements in the industry. However, only two out of 15 competencies were listed under ICT even though it is an upcoming domain. Thus, this study argues that more attention should be paid to digital competencies because the pandemic has accelerated the digitalization in logistics companies and the logistics professionals are ill-prepared (Gupta et al., 2022). Fortunately, Kohl et al. (2020) recently explained Industry 4.0's impacts on digital competencies, however, a more holistic competency framework is still needed as the impacts of COVID-19 do not stop on a technological level. COVID-19 has also restructured working environments by forcing professionals to work remotely and companies to implement e-business models (Kannan and Garad, 2021). Therefore, it is timely to consolidate and review the literature to comprehensively identify the emerging competencies, research trends on the topic, and update the competency frameworks so that logistics professionals are prepared for digitalization and can contribute to the competitiveness of the logistics industry.

Hence, the objective of this study is to review, identify and update the emerging competencies required by logistics professionals in the digital era. This review study will provide a state-of-the-art framework of emerging competencies by holistically including technical, digital, and personal competencies that are in line with Industry 4.0 and COVID-19. It can be used by logistics companies and institutes of higher learning as training and education recommendations to upskill aspiring logistics professionals and minimize the competency gap. Ultimately, logistics professionals can adapt to their work environments seamlessly and will be less fearful of losing their jobs due to incompetence.

The remaining sections are structured as follows. Section Methods discusses the relevant literature synthesized from Scopus. Section Results and discussion organizes the emerging competencies into their respective categories and discusses them individually. Section Conclusion is the conclusion, where implications, limitations, and recommendations are provided.

## Methods

### Preferred Reporting Items for Systematic Reviews and Meta-Analyses

**Figure 1:**
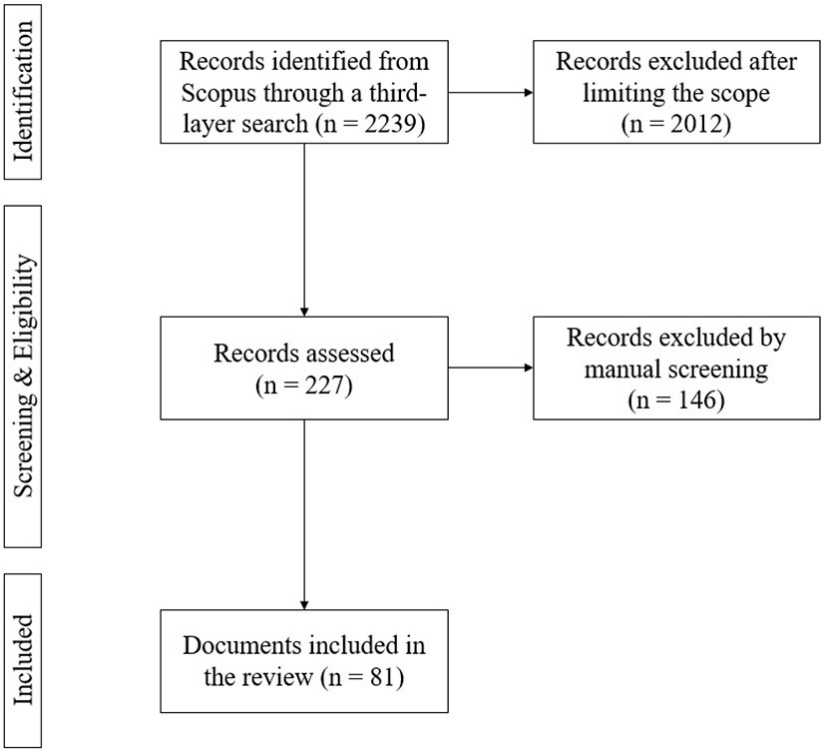
PRISMA flow diagram.

The chosen research method is a literature review as it aids in identifying and compiling the concepts highlighted in selected studies (Rowley and Slack, 2004), which will be useful when conceptualizing a state-of-the-art framework of emerging competencies for logistics professionals in the digital era. This study will follow the PRISMA guidelines for better accuracy and transparency of the review through a flow diagram consisting of identification, screening, eligibility, and inclusion, referenced from https://prisma-statement.org/PRISMAStatement/FlowDiagram. This is presented in Figure 1, and the phases will be elaborated on below.

#### Identification

In this phase, relevant academic documents are identified in databases through a multi-layer search on the title, abstract, and keywords. Numerous databases allow access to academic and conference publications, but this study has chosen Scopus as it is from the largest publishing house, Elsevier, and contains a comprehensive and curated abstract and citation database in areas such as business, management, and accounting.

Subsequently, a three-layer search of keywords was developed (Table 1). Asterisk was used to capture words in their singular and plural forms and words that had British and American English spellings. For example, using “capab^*^” would capture keywords such as “capable”, “capability,” and “capabilities.”

**Table 1:** Search keywords and results.

**Search keywords**	**Search results** ^**1**^	
	**Before**	**After**
**a) First-layer search structure**	1,448,350	27,886
industry 4.0* OR logistics 4.0 OR digital* OR ICT OR information and communication technolog*	
**b) Second-layer search structure**	225,251	7,852
industry 4.0* OR logistics 4.0 OR digital* OR ICT OR information and communication technolog	
AND	
competen* OR skill* OR knowledge OR abilit* OR talent* OR capab* OR proficien* OR aptitude	
**c) Third-layer search structure**	2,239	227
industry 4.0* OR logistics 4.0 OR digital* OR ICT OR information and communication technolog	
AND	
competen* OR skill* OR knowledge OR abilit* OR talent* OR capab* OR proficien* OR aptitude	
AND	
logistic*	

^1^Number of academic documents before and after limiting the search to Business, Management, and Accounting and the years 2016–2021.

The first-layer search contains keywords related to Industry 4.0. Although this is a substantial amount, not all relevant literature is captured as some may not specifically mention Industry 4.0. To expand the search, imperfect substitutes such as “logistics 4.0” and “information and communication technologies” were used. The second-layer search contains keywords related to the competencies of logistics professionals, whereby synonyms like “skills,” “knowledge,” and “abilities” are used in the search. The third-layer search includes “logistics” which filter out documents related to the topic.

Thereafter, a multi-layer search technique is performed, but it is limited to the title, abstract, and keywords for better accuracy and relevance of the search results. The scope of the study was also limited to “business, management, and accounting” and only includes studies published from 2016 to 2021. Doing so omits documents from less relevant fields and ensures that the emerging competencies from recent years can be captured.

This search was conducted on 5 December 2021, and Table 1 showcases the academic documents before and after limiting the subject areas and years. The first-layer search was performed and captured 27,886 academic documents related to Industry 4.0. Next, the second-layer search relating to competencies of logistics professionals captured 7,852 academic documents. Finally, the third-layer search was performed and captured 227 documents relevant to the logistics and supply chain sectors, and this is a good amount for manual screening of the documents.

#### Screening and eligibility

The 227 academic documents were then reviewed based on their relevance to emerging competencies for logistics professionals in the digital era. During the screening process, the academic documents were assessed based on the relevance of their titles and abstracts to the topic of this study i.e., emerging competencies for logistics professionals. Several documents were excluded as they focused on harnessing Industry 4.0's technologies for value creation in specific industries such as retail, agriculture, and healthcare, but did not discuss emerging competencies required by logistics professionals to provide these value-added services. Hence, they were not applicable to this study and were excluded.

The remaining academic documents were then checked for their eligibility, whereby they were assessed in full-text this time instead of solely based on their titles and abstracts. Many academic documents were focused on production and manufacturing activities. However, logistics is a subset of supply chain management and does not involve the manufacturing and production of goods from raw materials. It mainly focuses on activities such as transportation and storage. Hence, those academic documents have been excluded as well. Additionally, many documents gave narrative descriptions and did not specify the competencies required to complete certain logistics activities. Hence, they were excluded as well.

#### Included

After the screening and eligibility checks, 81 academic documents were retained. The characteristics of the 81 academic documents are collated in Table 2. It can be observed that the documents most relevant to this study are from the most recent years i.e., 2021 (*f* = 28) and 2020 (*f* = 19). Coincidentally, these years also coincide with the period where COVID-19 has accelerated digitalization and forced professionals worldwide to work remotely, which ultimately forces companies to relook at their business structures and required competencies in the workplace.

**Table 2:** Descriptive statistics (*n* = 81).

**Characteristics**	***f***
**Years of publication**	
2021 (as of 5 December 2021)	28
2020	19
2019	16
2018	13
2017	3
2016	2
**Source Title**	
International Journal of Logistics Management	5
Proceedings of the European Conference on Innovation and Entrepreneurship, ECIE	4
Journal of Enterprise Information Management	3
Journal of Modeling in Management	3
International Journal of Production Economics	2
Others	64
**Document Type**	
Article	56
Conference Paper	18
Review	5
Editorial	1
Book Chapter	1
**Publisher**	
Emerald	26
Elsevier	12
Institute of Electrical and Electronics Engineers Inc.	7
Taylor and Francis	5
Academic Conferences and Publishing International Limited	4
Others i.e., Wiley, Springer	27

The academic documents are credible as they are published by reputable publishers like Emerald (*f* = 26) and Elsevier (*f* = 12) which employ detailed review processes. The documents are published in different journals, with the more common ones being the *International Journal of Logistics Management* and *Proceedings of the European Conference on Innovation and Entrepreneurship*. Most are journal articles (*f* = 56) and conference papers (*f* = 18).

### Identify research themes

**Figure 2:**
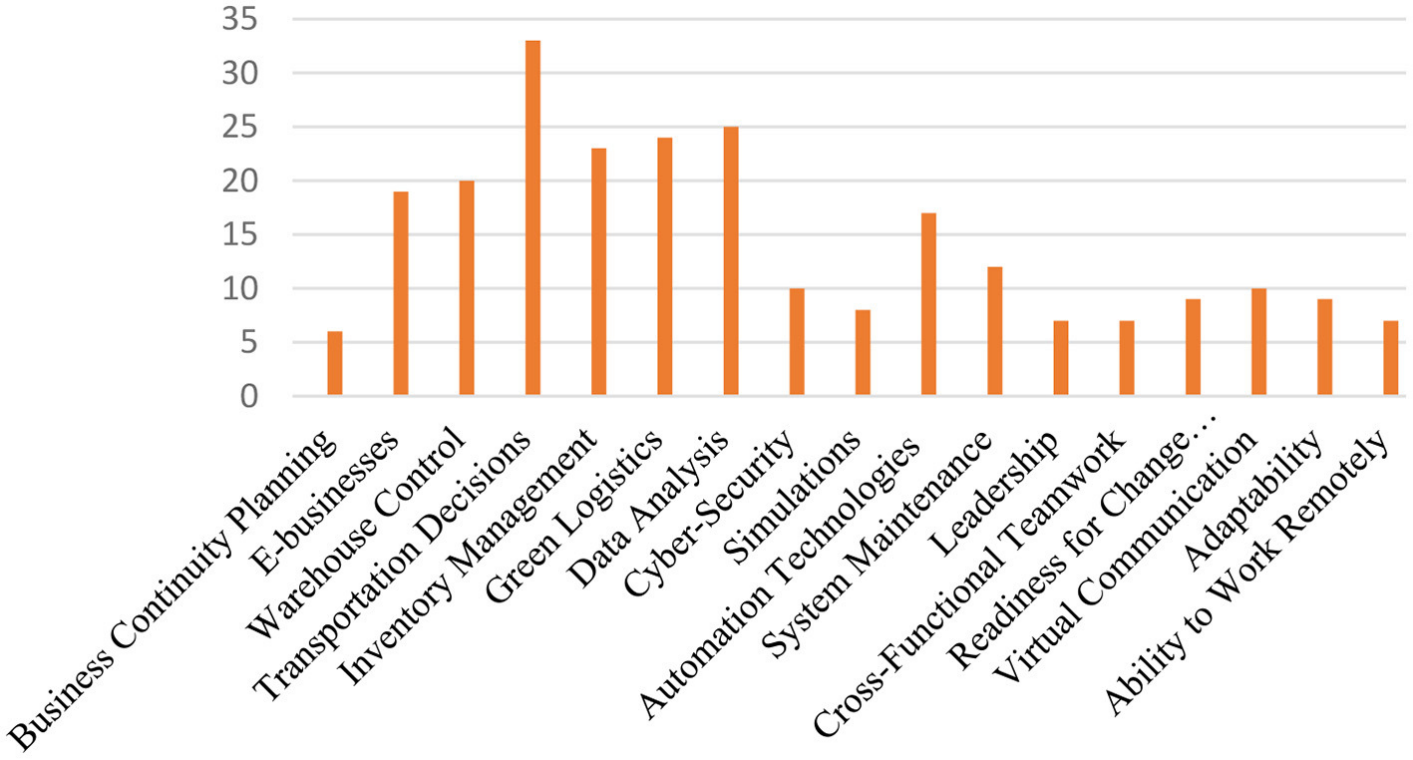
Distribution of academic documents for each sub-competency.

**Figure 3:**
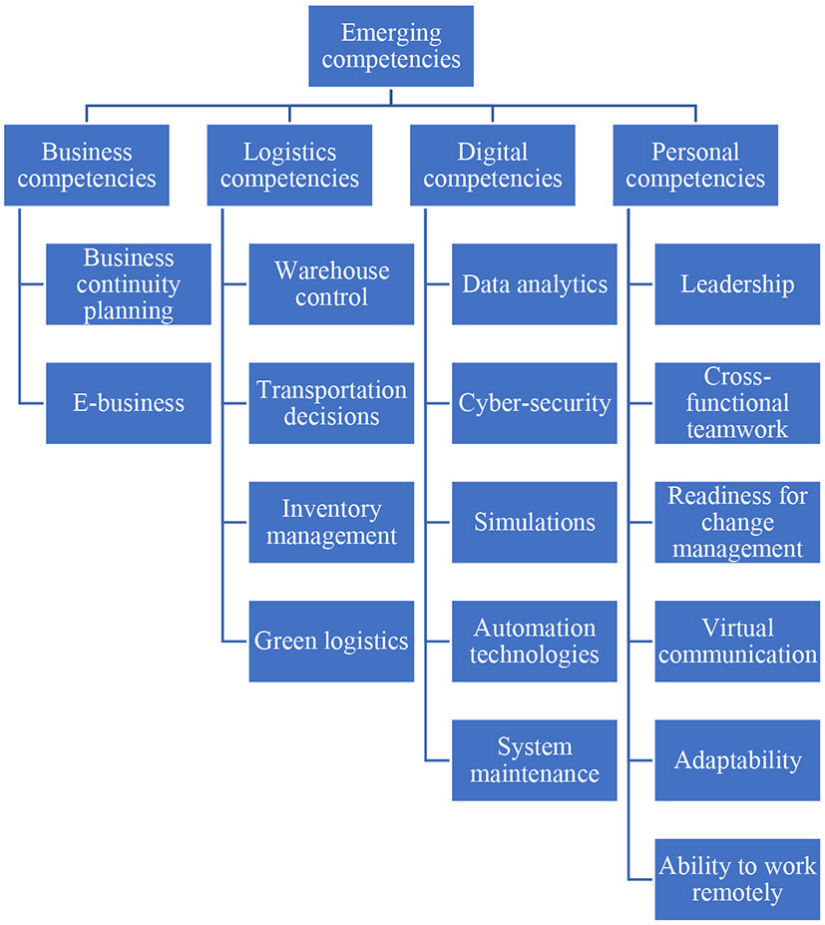
Emerging competencies framework.

The emerging competencies found in the 81 academic documents were identified and their distributions are shown in Figure 2. Thereafter, they were grouped into four main competencies: (1) business, (2) logistics, (3) digital, and (4) personal, as presented in Figure 3.

## Results and discussion

In this section, the four main competencies and their respective sub-competencies are discussed.

### Business competencies

In the wake of the disruptions caused by digitalization and COVID-19, resilience measures and e-business models are essential for adapting to these ever-changing environments. Hence, this section will explore these competencies.

#### Business continuity planning

During the COVID-19 pandemic, restrictive transportation policies, business closures, and port congestions have disrupted global transportation links and supply chains. Consequently, these COVID-19 measures affected the interconnectivity and density of trade, whereby a 1% increase in COVID-19 reduced global trade operations by 0.0948% (Khan et al., 2022). Hence, resilience is key and logistics professionals must monitor the uncertain business environment to reconfigure resources with agility (Ralston and Blackhurst, 2020).

To do so, Ivanov (2021) highlighted that logistics professionals must Design-for-Resilience by possessing strategic foresight for maintaining safety stock, subcontracting capacities, and backing up supply to cater to excessive and unexpected demand.

Secondly, logistics professionals must recognize the most vulnerable parts of the supply chain network and develop countermeasures to minimize disruptions and speed up recovery efforts (Zouari et al., 2021).

Thirdly, logistics professionals also need to be familiar with technologies for real-time monitoring and visibility (Ivanov, 2021). For instance, cloud computing and blockchain technologies provide enhanced flexibility in order fulfillment and efficiency to foster resilience (Zouari et al., 2021).

#### E-business

Internet and mobile technologies kickstarted the digital era and made the world more interconnected, thus businesses are transforming to e-business models with e-payments and e-signatures to maintain interfirm relationships (Kannan and Garad, 2021).

One aspect of successful e-business models is the logistics professionals' ability to handle the paradigm shift toward electronic business transactions (Sundaram et al., 2020). They should be able to utilize electronic telecommunication services to manage business processes from various operating areas as well as digitalize the downstream transactions with customers (Demirova, 2019; Gaudenzi et al., 2021).

Another aspect is logistics professionals' ability to go through unstructured digital channels such as social media, smartphone applications, and internet-based gadgets to sieve out intelligent enterprise strategic information, and apply charts, spreadsheets, modeling, and visualization tools to gather insights (Łobaziewicz, 2016). These insights enable professionals to craft information-driven strategies that align with the company's vision, mission, and objectives while achieving a robust business e-business framework.

### Logistics competencies

In recent years, there has been increasing integration of technologies in areas such as warehousing and transportation (Nitsche et al., 2021), and growing concern for sustainability among consumers. Hence, advanced capabilities in warehouse control, inventory management, transportation optimization, and green logistics are required for today's dynamic logistics environment.

#### Warehouse control

Good warehouse management ensures seamless material flow throughout the ever-increasing chains of shipment (Choudhury et al., 2021), and this is made possible with integrated warehouse controls and technology.

To improve the accuracy and efficiency of order-picking processes in warehouses, technologies such as voice-picking, automatic storage and retrieval systems, and automatic guided vehicles are implemented (Zijm and Klumpp, 2016; de Vass et al., 2020). Voice-picking uses voice instruction and recognition systems to direct logistics professionals while the automatic mechanisms direct machines (Lucas, 2021). Regardless of the technologies used, logistics professionals must be able to optimize internal material flow and routing (Choudhury et al., 2021). They should be able to direct the machines/professionals to pick up the right pallets from the right storage modules and deliver them to the assigned locations in the warehouse while minimalizing wasted movements in these simultaneous bulk workflows (Karunarathna et al., 2019).

Moreover, logistics professionals must be familiar with warehouse management tools such as bar-code scanners and camera-based scanners as they are utilized in receiving and returns, putaway, and packing (de Vass et al., 2020; Lucas, 2021). Logistics professionals should also be able to identify bottlenecks in the operations e.g., insufficient storage capacity, and utilize modern technologies such as 3D designing to replicate feasible warehouse space designs (Choudhury et al., 2021).

#### Transportation decisions

Reliable delivery services refer to delivering the right product in the right quantity to the right customer at the right place, time, and price (Salam, 2021). To enhance dispatch speed, accuracy, and cost-efficiency, RFID and IoT are used extensively in road transportation (Shah et al., 2020).

Logistics professionals should gather data using ICT technologies and apply them to optimize route planning and transportation scheduling (Suresh and Vasantha, 2018). They would have to practice dynamic routing that allows editing of routes because of logistics and environmental constraints before or during job executions, routing accuracy that requires logistics professionals to use routing engines to choose the most optimal route, and multi-routing planning as logistics professionals have to account for the different truck drivers, pitstops, and deliveries over various days (Möller et al., 2020).

Next, real-time tracking and tracing of vehicles and containers are also becoming the norm in the digital era (Suresh and Vasantha, 2018). There are technologies like GPS modules, NFC chips, barcodes, and RFID that provide real-time visibility and transparency of the transport cycle (Choudhury et al., 2021). By collecting real-time information on vehicle and cargo movements, logistics professionals can oversee and synchronize these movements over various transportation modes and organizations while reducing distribution attempts and lost products (Boschian and Paganelli, 2016).

New delivery methods like unmanned aerial vehicles and driverless cars are being experimented on (Wang et al., 2020). This calls for more coordination among delivery and supply chain stakeholders to upkeep good door-to-door services for customers (Suresh and Vasantha, 2018). Additionally, logistics professionals must be familiar with the existing and upcoming rules and regulations of these delivery methods for the areas they are operating in, as the newer delivery methods may be under strict regulations and allow operations in some countries only.

#### Inventory management

The inventory management process is also switching up to RFID technologies, the internet of things, and barcodes to circumnavigate stock and inventory management issues (Shah et al., 2020).

For example, data-intensive methods from Blue Yonder produce forecasts at the most granular levels e.g., forecasts of SKU levels for different points of sale, which logistics professionals can utilize to improve inventory accuracy and reduce obsolescence and stockouts (Choudhury et al., 2021). Additionally, with the usage of IoT and RFID technologies, information can be gathered and shared quickly and allows logistics professionals to further control stock movements, quantity, arrivals, and exits (Shah et al., 2020).

#### Green logistics

With the growing concerns over climate change, stringent environmental requirements have been established (Sharma et al., 2020). Hence, there is a paradigm shift toward a greener supply chain and logistics (Zijm and Klumpp, 2016), with more focus on sustainability and energy efficiency during operations to reduce environmental footprints (Pessot et al., 2021).

To achieve this, logistics professionals must be environmentally conscious and adopt sustainable practices (Reis et al., 2021). Logistics professionals should explore cleaner forms of energy and select the most feasible, cost-efficient, and environmentally friendly ones to facilitate the transition from traditional energy sources. Additionally, with blockchain, artificial intelligence, and cloud computing used extensively, logistics professionals can check the status of operations digitally, and reduce paperwork (Khan et al., 2022).

Next, Bag et al. (2021) highlight the importance of cultivating green teams based on continuous education about sustainability and implementing various training programs to share the best practices and transfer the knowledge. As such, these teams will be able to spread awareness about environmental sustainability and implement green logistics initiatives.

Green logistics is also important for sustainable manufacturing in the supply chain (Bag et al., 2021). Logistics professionals can push for modular product designs to increase package density and adopt bio-degradable packaging materials to reduce the environmental pollution per unit (Zijm and Klumpp, 2016).

Moreover, logistics professionals should strive for circular economies and closed-loop supply chains. With the Industry 4.0 technologies, they can access digital customer information to optimize their inventories as well as stop the manufacturers from overproducing for the forward supply chain (Sharma et al., 2020). As for reverse logistics, logistics professionals manage these returns and should send them to be recycled, reused, and remanufactured in the supply chain to close the loops and promote circular economies.

### Digital competencies

Industry 4.0's disruptive technologies are used extensively in the digital era. Hence, “digital thinking” has emerged as a new area of knowledge essential for logistics management processes (Kannan and Garad, 2021).

#### Data analytics

Supply chain applications have generated vast amounts of data through transactions and operations (Hallikas et al., 2021). However, data might be incomplete or biased, and technologies cannot think strategically, hence logistics professionals must utilize data mining software to properly interpret and assess the data and remain critical when extracting them (Schniederjans et al., 2020; Hallikas et al., 2021).

Supply chains also require accurate data collection and efficient information sharing among stakeholders to reduce demand uncertainties and improve supply chain visibility and collaboration (Adeitan et al., 2021). Consequently, logistics professionals must utilize intelligent algorithms to ensure that there are no inaccuracies in data collection and delays in information transfer (Liu et al., 2021). They must also align inter-firm processes and facilitate shared platforms for integrated upstream and downstream information sharing within the supply chain (Strategic Direction, 2021).

Following this, logistics professionals can utilize the information for decision-making and problem-solving (Chauhan et al., 2021). With the algorithms used for machine learning and deep learning, decisions and predictions can be made based on some pre-established rules (Lamdasni and Okar, 2020). Hence, logistics professionals can utilize analytics tools to evaluate the data and generate different solutions for different bottlenecks in the supply chain (Hallikas et al., 2021). This guides logistics professionals in decision-making and problem-solving processes.

#### Cyber-security

IoT and cloud computing allow extensive sharing of sensitive information and data for supply chain collaboration (Zissis, 2017). Unfortunately, doing so increases the risks of security breaches, network interceptions, and malware proliferation (Kannan and Garad, 2021). Thus, the cybersecurity framework from the National Institute of Standards and Technology emphasizes identifying and detecting threats and protecting, responding, and recovering from them (NIST, 2018).

To identify potential areas of cyber-attacks and data theft, logistics professionals must have technical knowledge about their company's online systems, hardware, software, and linked services (Nitsche et al., 2021). Logistics professionals must also continuously monitor anomalies to detect cybersecurity events and alert the IT personnel if there are suspicious activities or behaviors e.g., strange connections from unknown devices (Pajunen, 2017; NIST, 2018).

Cybersecurity awareness is also needed as the company's systems can be attacked in various ways. Pajunen (2017) highlights that phishing attempts can take place in e-mails, where the links redirect users to malicious sites. They can also occur *via* infected hardware and software, and when no in-house professionals monitor maintenance and installation works done by third-party technicians (Pajunen, 2017). Thus, logistics professionals must remain vigilant and check the systems before connecting to the company's main systems. They must also practice preventive maintenance routines like anti-virus software and develop contingency plans that can deal with the cyberattacks immediately should they occur.

#### Simulations

There is a shift toward strategic management concepts when designing and planning strategies in the digital era (Sharma et al., 2020). Logistics professionals can utilize simulations like virtual reality and augmented reality to visualize various scenarios before identifying the ideal transportation routes and warehouse layouts (Edirisuriya et al., 2018). Consequently, logistics professionals can optimize resources.

Smart glasses are becoming increasingly prevalent in warehouses (Schniederjans et al., 2020), and the demand is projected to hit USD 4.4 billion in 2022 (Rejeb et al., 2021). It is a wearable device equipped with sensors and GPS that augments the user's physical environment with virtual objects for interactive digital experiences (Schniederjans et al., 2020; Rejeb et al., 2021). With the smart glasses' enhanced information processing capabilities, logistics professionals should follow its commands regarding navigation and picking quantity to accelerate work and reduce mistakes (Wilkesmann and Wilkesmann, 2018; Rejeb et al., 2021).

#### Automation technologies

Automation technologies include sensors, robots, drones, and autonomous vehicles (Schniederjans et al., 2020). Logistics decisions such as the best transportation methods are made autonomously by these technologies (Wilkesmann and Wilkesmann, 2018). Logistics professionals must check that the deep learning algorithms for machine learning are meeting logistics needs (Kannan and Garad, 2021), e.g., ensuring that variabilities like order changes and seasonal or daily fluctuations are accounted for when planning capacity (Wilkesmann and Wilkesmann, 2018).

Additionally, the main component of automation is the cyber-physical system as it integrates all physical and information systems for worldwide access (Edirisuriya et al., 2018). Consequently, with the interdependent and intricate system landscape of software and hardware interactions, logistics professionals must conduct feasibility and compatibility assessments to ensure that the newly integrated automation technology complements the existing systems for guaranteed simultaneous usability in the logistics chains (Nitsche et al., 2021).

Wilkesmann and Wilkesmann (2018) emphasize that acceptance of these technologies is key. This is especially relevant during the COVID-19 pandemic, as the adoption of automation technologies has accelerated. Instead of fearing that they will lose their jobs to these technologies, logistics professionals must be open-minded to artificial intelligence and upgrade their skills for supervisory roles instead.

#### System maintenance

Technologies are bound to break down and require maintenance from time to time. When dealing with maintenance, Wilkesmann and Wilkesmann (2018) highlighted that troubleshooting, timely maintenance, and repair should be included in the learning processes.

To troubleshoot properly, logistics professionals should be familiar with a multitude of intelligent monitoring and measurement systems. There are real-time intelligent multiple fault diagnostic systems that make use of sensors, intelligent predictive decision support systems, and “Watchdog Agent” to process multiple failure analyses (Lamdasni and Okar, 2020). With real-time data modeling and simulation, logistics professionals can predict fault occurrences (Sharma and Joshi, 2020).

If faults occur, there are four categories of maintenance: (1) corrective maintenance, (2) preventive maintenance, (3) planned maintenance, and (4) condition-based maintenance (Sharma and Joshi, 2020). This means that logistics professionals must choose the optimal maintenance category to correct the faults while meeting customer service and business operations requirements. Hence, logistics professionals should also be familiar with the deep digital maintenance process as it utilizes profit loss indicators and neural analyses to optimize maintenance planning (Lamdasni and Okar, 2020). Alternatively, logistics professionals can also monitor and track product performance and utilize this information to assess the maintenance requirements for the technology (Kannan and Garad, 2021).

### Personal competencies

Other than hard skills, logistics professionals must also have management competencies that often include soft skills. Hence, this section will explore the upcoming personal competencies that logistics professionals should possess.

#### Leadership

Digital transformation and virtual work environments have emphasized the need for logistics companies to transition digitally. For this to happen, logistics managers must be open-minded about the digital strategies generated by professionals and be willing to invest in the required digital tools e.g., sensors, applications, and platforms (Hallikas et al., 2021).

They should also incorporate digital training and learning cultures to promote digital maturity in the workplace (Zouari et al., 2021). However, changing the work culture and employees' mindset is difficult, hence logistics professionals should foster digital readiness and passion by gradually implementing digitalization tools in different stages and rewarding those who participate actively (Lamdasni and Okar, 2020).

Unfortunately, some professionals may feel overwhelmed by these ever-changing internal and external business environments. Hence, logistics managers should adopt value-driven leadership styles over power-driven ones (Kannan and Garad, 2021), and possess psychological skills to support the physical and mental well being of logistics professionals by listening, supporting, and motivating them (Fenlon, 2020; Wehrle et al., 2020).

#### Cross-functional teamwork

Cross-functional teams perform purchasing, storage, and dispatching activities (Sandberg and Abrahamsson, 2011). Hence, cross-functional teamwork is vital and departments must cooperate and coordinate with each other. This promotes synchronization of inter-departmental activities, provision of value-added services, enhanced competitiveness, and better customer service (Ferreira et al., 2019).

Thus, the ability to work in cross-functional teams is important. This is a team performance, and professionals should not be trying to outdo each other. Instead, they must be team players who listen and communicate effectively.

The logistics professionals must also be agile and familiar with the job scopes of other departments so that they can respond to market changes quickly (Sandberg and Abrahamsson, 2011). Hence, they should be cooperative and share information resources to facilitate team decisions (Chen et al., 2018).

#### Readiness for change management

Logistics and supply chains experience frequent technological, social, and market evolutions (Klumpp and Zijm, 2019). To attain sustainable development, logistics companies must be prepared for change management (Thakur and Mangla, 2019). This is especially crucial in today's economy where digitalization has prompted for human-machine-interactions while the COVID-19 pandemic has disrupted global supply chains.

The digitalized and automated processes in logistics systems are changing traditional work processes by replacing manual work completed by logistics professionals. Hence, to cope with the different work processes, work tasks, and environmental conditions (Kuhlmann and Klumpp, 2017), logistics professionals must be ready for change management. They must be able to adapt to human-machine interactions while coping with the changing economic thinking, economic behavior, and economic mechanism caused by digitalization (Korchagina et al., 2020).

The pandemic has also forced logistics companies to relook at their business processes due to the constantly changing cost factors, dispatching arrangements, and supply circumstances (Hoek, 2020). To cope with supply shortages, logistics professionals should change supplier terms that favor suppliers better e.g., faster payments, or form collaborative relationships with suppliers for priority in their orders (van Hoek and Dobrzykowski, 2021). Logistics professionals also had to arrange for contactless deliveries. Hence, logistics professionals had to be resourceful, flexible, and willing to make changes to accommodate supply chain needs.

#### Virtual communication

In the digital era, there are various collaborative virtual software e.g., WhatsApp and Microsoft Teams that allow logistics professionals to communicate with global supply chain stakeholders without being in the same room (Kannan and Garad, 2021). When interacting with stakeholders from diverse cultures, logistics professionals should understand each other's cultures and establish a common working language to reduce conflicts, misconceptions, and inter-organizational uncertainties (Dethine et al., 2020).

Virtual communication has become even more relevant now with the introduction of e-commerce whereby online orders are from all over the globe. Furthermore, most professionals are working from home due to the pandemic. This means that face-to-face meetings are less viable, while virtual meetings, email correspondences, and phone communications are preferred (Chen et al., 2018). Logistics professionals will need to have more up-to-date computer skills such as screen-sharing, uploading documents in SharePoint for teammates to view, and directly editing work content together in Microsoft Teams (Gruenwald, 2021). They also require information technology skills for digital content creation in areas such as PowerPoint slides, word documents, and excel spreadsheets (Ariansyah et al., 2019).

#### Adaptability

We are living in a face-paced digital world where information is widely available and viewable through phone or tablet applications, forcing logistics professionals to adapt to work-life changes by being flexible in work and personal time arrangements (Kannan and Garad, 2021). Thus, adaptability and flexibility are key competencies that logistics professionals must possess and Chen et al. (2018) explained that this includes the professionals' learning adaptability and adaptability to industrial changes.

Learning adaptability is important as the half-life of knowledge is much shorter now in the fast-paced digital era (Wehrle et al., 2020), hence logistics professionals must be proactive in picking up new skills (Chen et al., 2018). They should be inquisitive, willing to learn on the job and pick up the skills quickly.

The COVID-19 pandemic has also shown that the future is unpredictable. Its far-reaching impacts have also forced many companies to invest in automation and implement work-from-home arrangements. Thus, the future is uncertain but logistics professionals must maintain a positive outlook and display adaptability to these changes (Chen et al., 2018). They should be self-reliant, calm, and take the initiative to update their knowledge and skills to handle the challenges.

#### Ability to work remotely

This trend of working remotely and virtually may continue as companies are venturing into automated systems by investing in robotics and artificial intelligence (Schniederjans et al., 2020).

In warehouses, automation technologies such as sensors and robots perform picking, storing, loading, and unl/oading warehouse functions while autonomous vehicles and drones can transport goods (Edirisuriya et al., 2018). As such, logistics professionals' physical presence is not required and they can work from home with office gadgets e.g., laptops and tablets. When doing so, they must know how to access the gadgets and their applications to monitor the logistics processes virtually and manage logistics processes remotely.

With working from home as the norm, logistics professionals have flexible working arrangements and hours (Kannan and Garad, 2021). However, this may make logistics professionals more prone to distractions and procrastination since nobody is around to monitor them. Logistics professionals must practice self-management by remaining on task and delivering quality work.

However, logistics professionals' work-life balance may also be compromised because by working virtually with the companies' gadgets, they are connected to the technologies and automated systems that provide them with real-time information and there will always be work to do (Kannan and Garad, 2021). Hence, logistics professionals must practice time management and strike the right balance such that their work-life does not into their private lives.

## Conclusion

In recent years, the logistics industry has been adapting to disruptions caused by Industry 4.0 and the pandemic. Industry 4.0 has pushed for technological advancements in logistics companies and COVID-19 has accelerated technological advancements e.g., robots to cope with safe-distancing regulations, resulting in a need to update digital competencies as well as look into how these technologies can be integrated into logistics operations i.e., logistics competencies. Furthermore, the pandemic has forced many logistics professionals to work remotely, which requires them to be open to change and adapt to new working environments quickly i.e., personal competencies. Companies were also required to have business continuity plans i.e., business competencies too. As such, there is a need to relook at the competencies required by logistics professionals in the digital era because existing competency frameworks such as the European Logistics Association Qualification Framework, humanitarian logistics competency framework, and BLM framework may not update the competencies required by logistics professionals in view of both Industry 4.0 and COVID-19. To address this gap, this study has adopted PRISMA guidelines and performed a third-layer search in Scopus that captured 227 documents relevant to the logistics and supply chain sector. Eighty one were retained after manual screening. Thereafter, this study analyzed, compiled, and categorized emerging competencies required by logistics professionals in the digital era. As presented in Figure 3, the framework is split into four domains: (1) business competencies to ensure continuity of operations, (2) logistics competencies to meet supply chain requirements, (3) digital competencies to adapt to the accelerated speed of digitalization caused by Industry 4.0 and COVID-19, and (4) personal competencies to handle digital and remote working environments. Overall, there are four key domains and 17 sub-domains.

This study has several contributions as well. Currently, existing literature about logistics professionals does not fully address how digitalization and COVID-19 are disrupting work environments and requiring new skillsets. Hence, the main contribution of this study is its state-of-the-art framework that specifically focuses on emerging competencies in the different domains. This was developed after analyzing existing literature on logistics professionals, compiling the emerging competencies required in the years of COVID-19 and digitalization, and subsequently organizing them into the framework consisting of competencies and sub-competencies.

Secondly, this study also paid equal attention to the different domains to ensure holistic coverage. As mentioned in the introduction, most existing literature mainly focused on a few domains e.g., business and logistics, and failed to provide a comprehensive analysis of the digital and personal competencies. Thus, the proposed framework has covered all grounds and presented the competencies clearly and objectively. Future studies can apply quantitative methods such as the fuzzy analytic hierarchy process to identify which competencies are deemed as most important by relevant stakeholders e.g., logistics companies.

Thirdly, the framework serves as a benchmark and can be used by human resource managers for skills upgrading. They can send logistics professionals who do not meet the benchmarks for in-house training or external training. This allows the human resource personnel to minimize competency gaps while allocating time and financial resources wisely. Additionally, the human resource managers can refer to the framework to craft interview questions when hiring new talents. Doing so verifies that the new talents are equipped with the required emerging competencies to contribute to their companies in the long run.

Forth, this framework can be utilized by Institutes of Higher Learning to refresh their curriculums by including the emerging competencies identified in this study. Hence, their curriculums are aligned with the logistics industry and will be more attractive to prospective students interested in the logistics industry. Additionally, graduates are also equipped with the relevant competencies required in the digital era and can integrate into the logistics companies seamlessly. Overall, the institutes will garner positive reviews and enjoy better ratings.

Despite the relevance of the emerging competencies framework, there are some limitations as well. Firstly, this study was focused on the emerging competencies required by logistics professionals in general. The professionals were not separated into different managerial positions e.g., senior logistics managers or entry-level logistics personnel. However, different managerial positions have different levels of competencies required, hence the generalisability of emerging competencies identified in this study is limited across the different managerial positions. Consequently, future studies about logistics competencies can limit their scopes to specific managerial positions for more comprehensive findings.

Secondly, this study addressed logistics professionals as a whole and did not split them by their age groups. However, older logistics professionals generally have lesser exposure to the digital world and may not be as receptive to the complex digital competencies compared to their younger counterparts e.g., entry-level logistics personnel. Thus, future research can interview older logistics professionals and ask them to score the four key domains and 17 sub-domains (identified in this study) based on their confidence levels. Thereafter, fuzzy analytic hierarchy process can be performed to identify those competencies that they are not confident in. This will allow future research to design a framework specifically for upskilling older logistics professionals as well as explore appropriate training methods to transition older logistics professionals into the digital era. Hence, logistics companies will be able to optimize resources for upskilling logistics professionals.

Another potential research direction is to further expand on the four key domains i.e., business, logistics, digital, and personal competencies by exploring their effects on organizational performance, employee satisfaction, knowledge integration capability, or organizational agility. This can be done by applying organizational learning theories, dynamic capabilities theory, transformative leadership theory, etc., and conducting structural equation modeling to confirm the interrelationships. By doing so, logistics companies will have a clearer visualization of strategic decisions to implement in the future.

## Data availability statement

The original contributions presented in the study are included in the article/supplementary material, further inquiries can be directed to the corresponding author.

## Author contributions

LK organized the database, performed the statistical analysis, and wrote the first draft and sections of the manuscript. All authors contributed to conception and design of the study, manuscript revision, read, and approved the submitted version.

## Conflict of interest

The authors declare that the research was conducted in the absence of any commercial or financial relationships that could be construed as a potential conflict of interest.

## Publisher's note

All claims expressed in this article are solely those of the authors and do not necessarily represent those of their affiliated organizations, or those of the publisher, the editors and the reviewers. Any product that may be evaluated in this article, or claim that may be made by its manufacturer, is not guaranteed or endorsed by the publisher.
